# Knockdown of piRNA pathway proteins results in enhanced Semliki Forest virus production in mosquito cells

**DOI:** 10.1099/vir.0.053850-0

**Published:** 2013-07

**Authors:** Esther Schnettler, Claire L. Donald, Stacey Human, Mick Watson, Ricky W. C. Siu, Melanie McFarlane, John K. Fazakerley, Alain Kohl, Rennos Fragkoudis

**Affiliations:** 1 The Roslin Institute and Royal (Dick) School of Veterinary Studies, University of Edinburgh, Easter Bush, Midlothian EH25 9RG, UK; 2 MRC-University of Glasgow Centre for Virus Research, 8 Church Street, Glasgow G11 5JR, UK; 3 The Pirbright Institute, Ash Road, Pirbright, Surrey GU24 0NF, UK; 4 ARK Genomics, The Roslin Institute, University of Edinburgh, Easter Bush, Midlothian EH25 9RG, UK

## Abstract

The exogenous siRNA pathway is important in restricting arbovirus infection in mosquitoes. Less is known about the role of the PIWI-interacting RNA pathway, or piRNA pathway, in antiviral responses. Viral piRNA-like molecules have recently been described following infection of mosquitoes and derived cell lines with several arboviruses. The piRNA pathway has thus been suggested to function as an additional small RNA-mediated antiviral response to the known infection-induced siRNA response. Here we show that piRNA-like molecules are produced following infection with the naturally mosquito-borne Semliki Forest virus in mosquito cell lines. We show that knockdown of piRNA pathway proteins enhances the replication of this arbovirus and defines the contribution of piRNA pathway effectors, thus characterizing the antiviral properties of the piRNA pathway. In conclusion, arbovirus infection can trigger the piRNA pathway in mosquito cells, and knockdown of piRNA proteins enhances virus production.

## Introduction

Arboviruses are unique in that they must naturally replicate in both their invertebrate vector and vertebrate host and are therefore subjected to the selective pressure of very different antiviral responses. One of the major antiviral responses in invertebrates is the RNA silencing pathway or RNA interference (RNAi). It has been shown that the RNAi pathway, in particular the exogenous small interfering (si)RNA pathway, is able to inhibit and restrict arbovirus infections in whole mosquitoes or mosquito cells (Blair, 2011; Donald *et al.*, 2012). The exogenous RNAi pathway is induced by virus-derived dsRNA that is recognized by a Dicer protein, Dcr-2, and is processed into 21 bp-long virus-derived siRNAs, also called viRNAs. After viRNAs are incorporated and unwound in the RNA-induced silencing complex (RISC) that harbours Argonaute 2 (Ago 2) as a catalytic domain, one strand of the viRNA is retained and used as a guide to find complementary viral RNA, which is then degraded. Until recently, it was believed that the anti-viral response in invertebrates is only attributed to the exogenous siRNA pathway. Recently, however, the PIWI-interacting RNA (piRNA) pathway has also been suggested to display antiviral activity. piRNA molecules differ from siRNAs in several aspects; they are produced by a Dicer-independent pathway; have a broader size range of 25−29 nt; are associated with proteins of the PIWI clade and have a so-called ‘ping-pong’ signature due to their production pathway, which is represented by a bias for U at position 1 in antisense piRNAs and A at position 10 in sense piRNAs (Saito & Siomi, 2010; Senti & Brennecke, 2010; Siomi *et al.*, 2010, 2011</xref>; van Rij & Berezikov, 2009). In *Drosophila melanogaster*, it has been shown that PIWI proteins are mainly expressed in germline cells and are thought to protect the germline from transposable elements by targeting the transcribed RNA of active transposons. However, PIWI proteins have also been detected in somatic cells (Brennecke *et al.*, 2007). Although their induction pathway is still not completely understood, two mechanisms have been proposed to describe piRNA biogenesis. Primary piRNA molecules are antisense to the genomic regions of transposons and derive from long precursor ssRNA that targets transposon-derived sense RNA. Upon cleavage, they give rise to secondary piRNA molecules that are mostly sense with an A_10_ bias. Secondary piRNAs are incorporated into Argonaute 3 (Ago 3) protein, which uses these piRNAs to find complementary antisense RNA, which again results in the production of primary-type piRNAs. This so-called ping-pong mechanism results in the generation of anti-sense primary piRNA molecules with a U_1_ bias. Primary piRNA molecules have mostly been found to form complexes with Aubergine (Aub) and PIWI proteins (Saito & Siomi, 2010; Senti & Brennecke, 2010; Siomi *et al.*, 2010, 2011</xref>; van Rij & Berezikov, 2009).

The detection of virus-specific piRNA molecules in drosophila ovary somatic sheet (OSS) cells was the first report suggesting that the piRNA pathway targeted viruses in insects (Wu *et al.*, 2010). More recently, virus-specific piRNA molecules have been reported in aedine mosquitoes for chikungunya virus (CHIKV) (*Togaviridae*, *Alphavirus*) (*Aedes albopictus* and *Ae. aegypti*) and dengue virus (DENV) (*Flaviviridae*, *Flavivirus*) (*Ae. aegypti*), and their derived cell lines can become infected with Sindbis virus (SINV) (*Togaviridae*, *Alphavirus*), La Crosse virus (LACV) (*Bunyaviridae*, *Orthobunyavirus*), Rift Valley fever virus (RVFV) (*Bunyaviridae*, *Phlebovirus*) and Schmallenberg virus (SBV) (*Bunyaviridae*, *Orthobunyavirus*) (Hess *et al.*, 2011; Léger *et al.*, 2013; Morazzani *et al.*, 2012; Schnettler *et al.*, 2013; Vodovar *et al.*, 2012). It is not known whether these virus-specific piRNA molecules actually mediate any antiviral activities or which proteins of the piRNA pathway are important for this response. The PIWI protein clade shows an expansion in aedine mosquitoes compared to drosophila, which is consistent with a role besides transposon targeting. *Ae. aegypti* encode seven Piwi proteins (Piwi 1, AAEL008076; Piwi 2, AAEL008098; Piwi 3, AAEL013692; Piwi 4, AAEL007698; Piwi 5, AAEL013233; Piwi 6, AAEL013227; Piwi 7, AAEL006287) and one Ago 3 protein (AAEL007823), compared to *D. melanogaster*, which only encodes one of each of Piwi, Ago 3 and Aub (Campbell *et al.*, 2008a).

Although expression of some of the PIWI proteins has been recently reported in *Ae. aegypti*-derived Aag2 cell lines (Vodovar *et al.*, 2012) and in the head and thorax of *Ae. albopictus* (Morazzani *et al.*, 2012), nothing is known about their involvement in antiviral activity. If the piRNA pathway acts as an antiviral response, then it would be expected that silencing proteins involved would have a positive effect on arbovirus replication as observed for the Ago 2 protein, which is known to be involved in the siRNA-based antiviral RNAi response (Campbell *et al.*, 2008b; Sánchez-Vargas *et al.*, 2009). To test this hypothesis, we investigated the importance of piRNA-related proteins on viral infection. Re-analysis of previous deep-sequencing data from mosquito-borne Semliki Forest virus (SFV) (*Togaviridae*, *Alphavirus*) infection of U4.4 (derived from *Ae. albopictus*) or Aag2 (derived from *Ae. aegypti*) cells (Siu *et al.*, 2011) revealed the presence of piRNA-like small RNAs mapping mainly to a section of the SFV genome, which decreased in Aag2 cells following knockdown for all Piwi/Ago 3 proteins. Silencing of PIWI 4 protein increased SFV replication and production but did not decrease the presence of SFV-specific piRNA-like molecules, confirming that the piRNA pathway does indeed display antiviral activity and that Piwi 4 possibly acts as an antiviral effector protein in this pathway.

## Results

### SFV-specific piRNA-like molecules in aedine cell lines

**Fig. 1.**
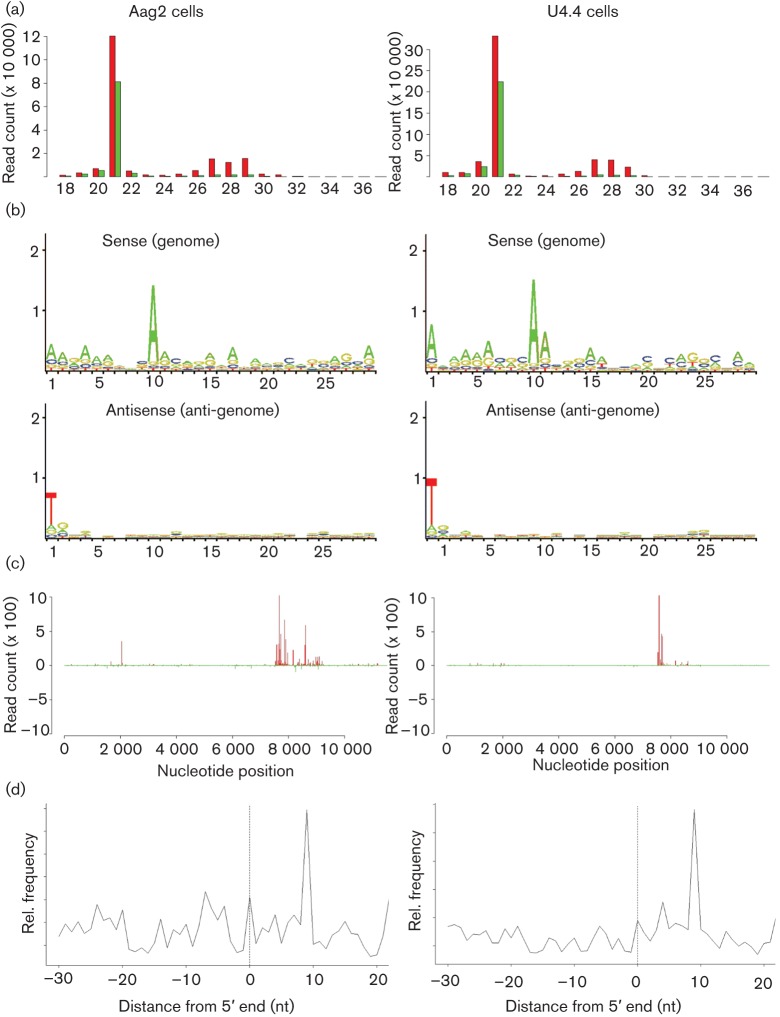
Aag2 and U4.4 cells produce both viRNAs and piRNA-like RNAs following SFV infection. (a) Size distribution of small RNA molecules mapping to the SFV genome or anti-genome in *Ae. aegypti* (Aag2) or *Ae. albopictus* (U4.4); RNA was isolated at 24 h p.i. Red and green indicate small RNAs mapping to the genome and anti-genome, respectively. (b) Relative nt frequency and conservation per position of 25–29 nt small RNAs mapping to the genome and anti-genome of SFV in Aag2 and U4.4 cells are indicated. Sequence is represented as DNA. The overall height of the nt represents sequence conservation. (c) Frequency distribution of 28 nt small RNA molecules to the SFV genome or anti-genome in Aag2 and U4.4. The *y*-axis shows the frequency of the 28 nt small RNAs mapping to the corresponding nt position of the *x*-axis (SFV genome length). Positive numbers represent the frequency of small RNAs mapping to the genome and negative numbers those mapping to the anti-genome. (d) Frequency map of 24–30 nt small RNAs mapping to the opposite strand of SFV4. Probabilities of complementarities of the sense and antisense SFV-specific small RNAs were mapped along the small RNAs (position 0 represents the first nt).

To determine whether the piRNA pathway specifically targets SFV in mosquito cells, we first investigated if these cells produce viral-specific piRNA-like molecules following infection. We re-analysed data previously obtained from deep sequencing of Aag2 and U4.4 cells infected with SFV [RNA isolation 24 h post-infection (p.i.)]; deep sequencing by using the Illumina Solexa platform as described in Methods (Siu *et al.*, 2011) and this time also mapped small RNAs greater than 26 nt in length to the SFV genome. As previously reported, the major species of virus-specific small RNA molecules were viRNAs 21 nt in length (Siu *et al.*, 2011); however, small RNAs mapping to SFV in the range of 25–29 nt could be observed for both cell lines (Fig. 1a). Most of these small RNA molecules mapped to the sense orientation of the SFV in the 5′ end of the subgenomic RNA and had a bias for A at position 10, a characteristic of secondary piRNAs (Fig. 1b and c). Besides, the 5′ ends of these complementary SFV-specific RNAs were most frequently separated by 10 nt, a feature of piRNAs produced by the ping-pong mechanism (Fig. 1d) This is consistent with what has previously been reported for SINV, CHIKV and SBV-specific piRNA-like RNAs (Morazzani *et al.*, 2012; Schnettler *et al.*, 2013; Vodovar *et al.*, 2012). Having demonstrated the production of piRNA-like RNAs in our mosquito cell infection systems, we proceeded to investigate piRNA pathway functionality by determining the effect of Piwi/Ago 3 silencing on SFV replication.

### Expression and knockdown of PIWI transcripts in Aag2 cells

**Fig. 2.**
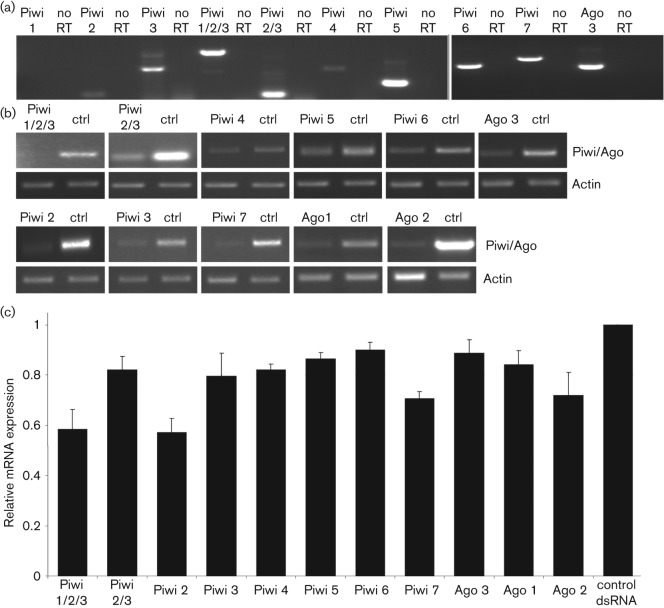
Expression and knockdown of piRNA-related transcripts in Aag2 cells. (a) Detection of Piwi (1/2/3, 2/3, 2, 3, 4, 5, 6, 7) and Ago 3 transcripts in *Ae. aegypti*-derived Aag2 cells by RT-PCR using oligo-dT primers for reverse transcription, and gene-specific primers for PCR. no RT represents the PCR product derived from samples lacking the superscript III enzyme. (b) dsRNA-based silencing of Piwi (1/2/3, 2/3, 2, 3, 4, 5, 6 and 7), Ago 3, Ago 1 and Ago 2 transcripts or cells transfected with eGFP-specific control dsRNA (ctrl) were detected in Aag2 cells by RT-PCR using gene-specific primers. Actin PCR product was used as a control. (c) Quantification of mRNA knockdowns using ImageJ software (National Institutes of Health). Graph shows the mean expression of five repeats normalized to actin expression and relative to eGFP-dsRNA controls. Error bars show standard errors of means.

Given the lack of genomic information for *Ae. albopictus*, these experiments were performed in *Ae. aegypti*-derived Aag2 cells. To produce dsRNA molecules specifically targeting single PIWIs or Ago 3, primers were designed to amplify unique regions of these genes by RT-PCR. Ago 2 depletion was taken as a positive control as it has been previously reported to be involved in the antiviral siRNA pathway (Campbell *et al.*, 2008b; Sánchez-Vargas *et al.*, 2009; van Rij *et al.*, 2006), and Ago 1 was a negative control that is known to be involved in the microRNA pathway. First, the primers were tested for their specificity to amplify unique regions of the Piwi/Ago 3 mRNAs. As previously reported (Vodovar *et al.*, 2012), we amplified Piwi 4, 5, 6 and 7, as well as Ago 3. Piwi 1–3 are highly homologous, making unique primer design difficult. Primers amplifying parts shared by either Piwi 1, 2 and 3 or only 2 and 3 were successful, as well as Piwi 2 and 3 alone; however, attempts to amplify a unique region of Piwi 1 were unsuccessful (Fig. 2a). Sequencing of the PCR products confirmed their origin. Before, silencing the Piwi and Ago 3 with the dsRNA produced by *in vitro* transcription, transfection efficiency of dsRNA in Aag2 was assessed and optimized using internally labelled fluorescent dsRNA molecules. A maximum of 28.6 % positive cells was observed (Fig. S1a, available in JGV Online). Cells were transfected with 100 ng dsRNA, either Piwi specific (1/2/3, 2/3, 2, 3, 4, 5, 6, 7 and Ago 3) or control (eGFP specific), at 24 h post-seeding using Lipofectamine 2000. Silencing of target transcripts was determined by semi-quantitative reverse transcriptase PCR (RT-PCR) 24 h post-transfection (p.t.), and several experiments were quantified in relation to control dsRNA using actin as a loading control (Fig. 2c). Aag2 cells treated with dsRNA specific for Piwi 1/2/3, 2/3, 4, 5, 6 and Ago 3 showed a 10–42 % reduction in target transcripts compared to controls treated with eGFP dsRNA. Similar results were observed for Piwi 2, 3 and 7 (Fig. 2b, c). A cell viability assay (cellTiter-Glo, Promega) was performed on all dsRNA-treated cells to determine whether transcript knockdown had an effect on cell viability, but no deleterious effect was observed (data not shown).

### Effect of Piwi/Ago 3 knockdown on SFV replication

**Fig. 3.**
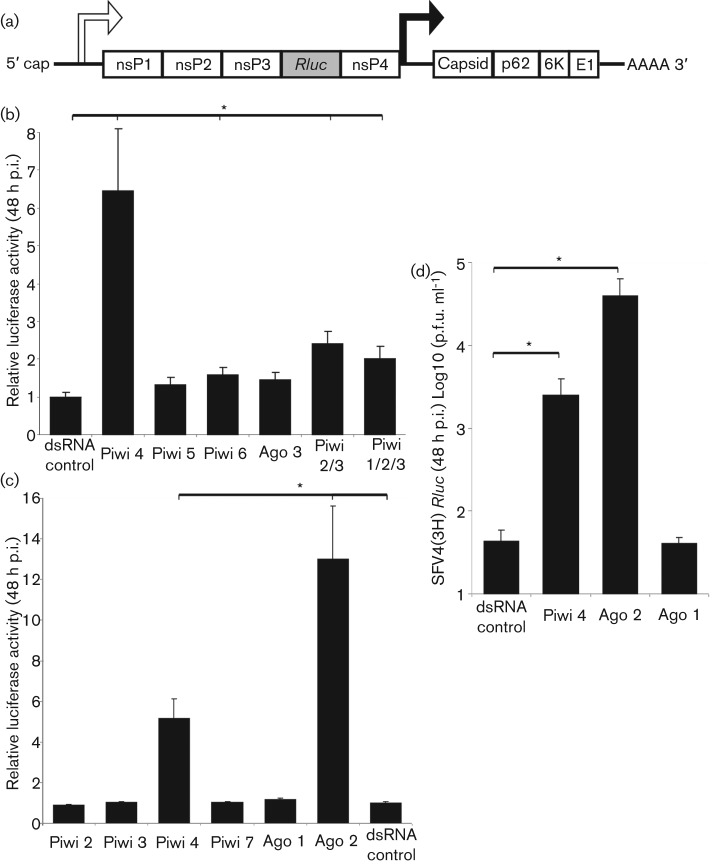
Piwi/Ago 3 proteins inhibit SFV replication in Aag2 cells. (a) Schematic representation of SFV4 encoding *Renilla* luciferase (*RLuc*) as reporter (flanked by duplicated nsP2-protease cleavage sites at the nsP3/4 junction) as part of the viral non-structural polyprotein; SFV4(3H)-*RLuc* virus. (b) Aag2 cells transfected with dsRNA against Piwi (1/2/3, 2/3, 4, 5 and 6), Ago 3 or eGFP-specific dsRNA (control) were infected with SFV4(3H)-*RLuc* 24 h p.t. at an m.o.i. of 0.1. The mean of four independent experiments performed in triplicate are shown with standard errors (* represents *p*<0.05, *t*-test). (c) As (b) with dsRNA specific against Piwi (2, 3, 4 and 7), Ago 2, Ago 1 or eGFP-specific dsRNA (control). Luciferase activity was measured 48 h p.i., and the means with standard errors are shown for three independent experiments performed in triplicate (* represents *p*<0.05, *t*-test). (d) SFV titre (p.f.u. ml^−1^) in supernatant of Piwi 4-, Ago 1- or Ago 2-silenced cells versus control (eGFP dsRNA) infected with an m.o.i. of 0.1 was determined 48 h p.i. by plaque assay. The means of three independent experiments performed in triplicate are shown with standard errors (* represents *p*<0.05, *t*-test).

Next, the effect of Piwi/Ago 3 silencing on SFV replication was investigated and compared to the knockdowns of Ago 1 and 2. dsRNA transfections in Aag2 cells were repeated, and at 24 h p.t., these cells were infected with the reporter alphavirus SFV4(3H)-*RLuc* [expressing *Renilla* luciferase (*RLuc*) as a replication marker] (Fig. 3a). Infections were performed at an m.o.i. of 0.1 (Fig. 3b, c), and *RLuc* activity was determined 48 h p.i. Significantly higher luciferase activity was detected in cells treated with Piwi 4-specific dsRNA compared to control (Fig. 3b, c). Cells treated with Piwi 6-, Piwi 2/3- and Piwi 1/2/3-specific dsRNA showed an increase in *RLuc* activity, although Piwi 4-specific dsRNA had a stronger effect (Fig. 3b). Knockdown of Ago 1 had no effect on luciferase expression compared to Ago 2 knockdowns, which exhibited the highest increase in luciferase activity (Fig. 3c). In addition, plaque assays performed with supernatant from dsRNA-transfected (eGFP, Piwi 4, Ago 1 and Ago 2) and SFV4(3H)-*RLuc*-infected cells (m.o.i. of 0.1) showed higher virus titre in cells treated with Piwi 4 or Ago 2 dsRNA (Fig. 3d). To ensure that the observed increase of *RLuc* activity in cells transfected with Piwi 4-specific dsRNA was not due to off-target effects of the dsRNA, experiments were repeated with two additional Piwi 4-specific dsRNA molecules (Piwi 4-2 and Piwi 4-3), resulting in similar *RLuc* activity (Fig. S1b). Overall, these results show that silencing Ago 2 and some Piwi, in particular Piwi 4, in Aag2 cells enhances SFV replication and virion production.

### Effect of PIWI/Ago 3 knockdown on the production of SFV-specific piRNA-like molecules

**Fig. 4.**
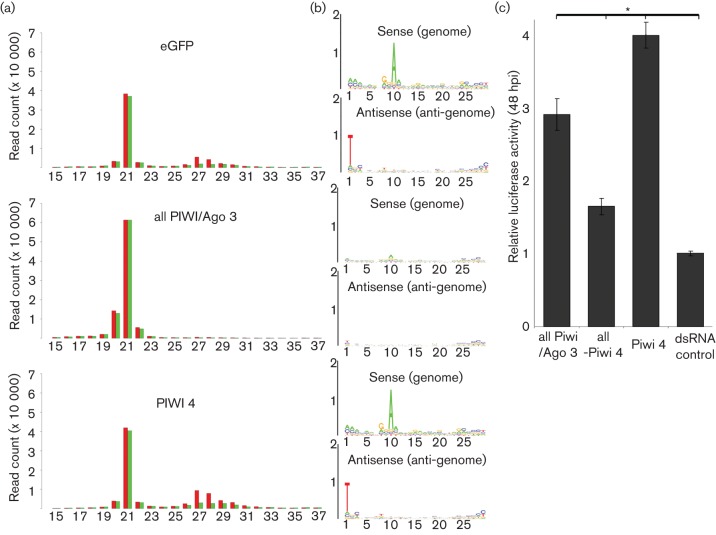
Piwi/Ago 3 proteins are involved in the production of SFV-specific piRNA-like molecules in Aag2 cells. (a) Size distribution of small RNA molecules mapping to the SFV genome or anti-genome in Aag2 transfected with eGFP-specific control dsRNA, a combination of Piwi/Ago 3 dsRNA (Piwi 1/2/3, 2/3, 2, 3, 4, 5, 6, 7 and Ago 3) or Piwi 4-specific dsRNA, followed by SFV4 infection; RNA was isolated at 24 h.p.i. Red and green indicate small RNAs mapping to the genome and anti-genome, respectively. (b) Relative nt frequency and conservation per position of 25–29 nt small RNAs mapping to the genome and anti-genome of SFV in Aag2 prior to transfection with the above-mentioned dsRNA molecules. Sequence is represented as DNA. The overall height of the nucleotide represents sequence conservation. (c) Aag2 cells transfected with different combinations of dsRNA (all Piwi/Ago 3: Piwi1/2/3, 4, 5, 6, 7 and Ago 3; all Piwi 4: Piwi1/2/3, 5, 6, 7 and Ago 3; Piwi 4 or dsRNA control: eGFP specific) were infected with SFV4(3H)-*RLuc* 24 h p.t. at an m.o.i. of 0.1. The means of three independent experiments performed in triplicate are shown with standard errors (* represents *p*<0.05, *t*-test).

Deep-sequencing experiments were performed to determine in more detail if Piwi 4 is needed for the production of SFV-specific piRNA-like molecules or rather acts as an effector molecule using the produced SFV-specific piRNA-like molecules to target the viral RNAs. Knockdown of all Piwi/Ago 3 proteins was performed to determined that any of these proteins are needed for the production of SFV-specific piRNA-like molecules. First, we established that the same cells could be targeted by dsRNA transfection and SFV infection using internally labelled fluorescent dsRNA and immunostaining for SFV nsP3 (Fig. S1a). Next, cells were transfected either with a combination of dsRNA molecules (targeting Piwi 1-3, 4, 5, 6, 7 and Ago 3) or Piwi 4-specific dsRNA alone, followed by SFV4 infection at an m.o.i. of 10. Cells transfected with eGFP-specific dsRNA were used as control. At 24 h p.i., total RNA was isolated, small RNAs were sequenced and the frequencies and SFV genome location of small RNAs were determined. All samples showed the presence of 21 nt SFV-specific small RNAs with a similar frequency to the genome and anti-genome; however, their frequency differs depending on the transfected dsRNAs, giving the highest number in cells transfected with a combination of piRNA/Ago 3-specific dsRNA, followed by Piwi 4-specific dsRNA, with control eGFP-specific dsRNA giving the lowest frequency. In addition, SFV-specific small RNAs of length 26–30 nt with a peak at 27 nt, mapping mainly to the sense orientation, could be observed in cells transfected with eGFP-specific control dsRNA and Piwi 4-specific dsRNA. Similar molecules were also present in cells transfected with a combination of Piwi/Ago 3-specific dsRNA but at a much lower frequency. These molecules have all the piRNA-specific features described for the previously identified SFV-specific small RNA molecules: characterized by an A_10_ bias in the sense molecules, a U_1_ bias in the antisense molecules (Fig. 4a, b) and separation of 10 nt of the 5′ends of the complementary small RNAs (Fig. S2b). As already observed for the other SFV-specific piRNA-like molecules, they mainly map to the 5′ end of the subgenomic RNA (Fig. 2a). To further characterize the response of SFV replication in these knockdown cells, the experiments were repeated following infection with the SFV4(3H)-*RLuc* reporter virus. Cells transfected with a combination of Piwi/Ago 3-specific dsRNA molecules, lacking Piwi 4 dsRNA, were also included. Increase in *RLuc* activity compared to control cells could be observed for all knockdowns; however, the strongest increase was present in cells with Piwi 4 knockdown followed by knockdown of all Piwi/Ago 3. Interestingly, cells transfected with a combination of Piwi/Ago 3-specific dsRNA but lacking Piwi 4-specific dsRNAs resulted in the lowest *RLuc* increase (Fig. 4c). Overall, these results support the involvement of Piwi/Ago 3 for the production of the SFV-specific piRNA-like molecules and suggest that Piwi 4 acts as an effector protein that targets the virus but is not needed for the production of SFV-specific piRNA-like molecules.

### Can dsRNA molecules induce piRNA production

**Fig. 5.**
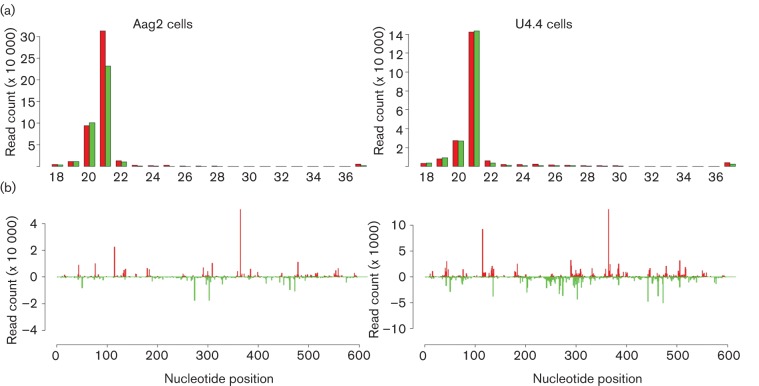
Transfected dsRNA feeds into the siRNA but not the piRNA pathway. (a) Size distribution of small RNA molecules mapping to the input eGFP sequence following transfection of eGFP-derived dsRNA (720 nt) into *Ae. aegypti* (Aag2) or *Ae. albopictus* (U4.4)-derived cells. RNA was isolated at 24 h p.t.. Red and green colour maps show the coding strand and non-coding strand of the input dsRNA, respectively. (b) Frequency distribution of 21 nt small RNA molecules to the eGFP coding strand (positive) or non-coding (negative) strand in Aag2 and U4.4. The *y*-axis shows the frequency of the 21 nt small RNAs mapping to the corresponding nt position of the 720 nt eGFP-specific dsRNA of the *x*-axis.

Knockdown experiments of Ago 3 performed in *Anopheles gambiae* suggests that at least some of the PIWI pathway proteins are also involved in the exogenous dsRNA-induced silencing response (Hoa *et al.*, 2003). In addition, recent experiments in aedine mosquitoes infected with transgenic CHIKV expressing the dsRNA-binding protein B2 suggest that dsRNA molecules are an inducer of the piRNA pathway (Morazzani *et al.*, 2012), as is known for the exogenous siRNA pathway. To investigate if dsRNA on its own can be processed into piRNA-like molecules, we transfected Aag2 or U4.4 cells with dsRNA molecules derived from the eGFP sequence. Subsequently, RNA was isolated 24 h p.t., followed by sequencing and mapping of small RNAs to the eGFP target sequence as described above. As expected, small RNAs of 21 nt in size that mapped to the sense or antisense orientation and along the eGFP sequence were observed as the majority, indicating induction of the exogenous RNAi pathway (Fig. 5a, b). Some small RNAs in the 25–29 nt range mapping to the eGFP input sequence were identified; however, no specific sequence logo identifying them as piRNA-like molecules was detected (data not shown). This suggests that ssRNA (for example from virus replication) is needed for the production of piRNA molecules but does not rule out a link between the siRNA and piRNA pathways.

## Discussion

Until now, antiviral RNA silencing activities in mosquitoes have mainly been reported for the exogenous siRNA pathway. The identification of piRNA-like virus-specific RNA molecules in drosophila OSS (Wu *et al.*, 2010), mosquitoes and mosquito-derived cells against different arboviruses suggested a contribution of the piRNA pathway in the antiviral response (Hess *et al.*, 2011; Léger *et al.*, 2012; Morazzani *et al.*, 2012; Schnettler *et al.*, 2013; Scott *et al.*, 2010; Vodovar *et al.*, 2012). However, this role has not been experimentally proven. The piRNA pathway is known to target transposons and thereby ensures genome stability, especially in germline cells. As some arboviruses have been reported to be vertically transmitted (Anderson *et al.*, 2012; Mulyatno *et al.*, 2012), an antiviral response by the piRNA pathway in germline cells may constitute an antiviral mechanism to inhibit vertical transmission or limit virus replication in developing embryos. On the other hand, a putative piRNA pathway in somatic tissues could add another layer to small RNA-based antiviral responses controlling arboviral infection. The finding that SFV-produced piRNA-like small RNA molecules in *Ae. aegypti-* and *Ae. albopictus*-derived cell lines is in accordance with recently published work showing similar results for CHIKV, SINV, LACV and SBV (Morazzani *et al.*, 2012; Schnettler *et al.*, 2013; Vodovar *et al.*, 2012). The observation that the knockdown of some PIWI proteins in Aag2 cells has a positive effect on SFV infection supports the hypothesis that the piRNA pathway and possibly the viral-specific piRNA-like small RNAs have an antiviral function in these cells. A similar result has been previously reported in anopheline mosquitoes. Indeed, *A. gambiae* showed an increase in O’nyong-nyong virus (*Togaviridae; Alphavirus*) following Ago 3 knockdown (Keene *et al.*, 2004). We extend this finding to aedine mosquitoes and highlight the additional contribution of Piwi 4. The fact that virus-specific piRNA-like small RNA molecules are not specific to *Ae. aegypti* but can also be found in infected *Ae. albopictus*, coupled with the expression of all piRNA pathway proteins (PIWIs and Ago 3) in somatic tissues (head and thorax) (Morazzani *et al.*, 2012), indicates that the ‘antiviral’ piRNA pathway is probably not specific to *Ae. aegypti* but could possibly be present in *Ae. albopictus* as well. It is not known if the same is true for drosophila. Viral-specific piRNAs have been described in drosophila OSS (Wu *et al.*, 2010), but it is not known if they have any antiviral activity in these cells. In addition, no viral-specific piRNAs have been reported in somatic tissue or derived cells of drosophila until now, which is in contrast to aedine mosquitoes and their derived cells (Hess *et al.*, 2011; Léger *et al.*, 2012; Morazzani *et al.*, 2012; Schnettler *et al.*, 2013; Vodovar *et al.*, 2012). This could be due to the differences in PIWI pathway protein expression between drosophila and *Ae. aegypti* (Campbell *et al.*, 2008a). However, knockdown of Piwi in drosophila results in increased WNV production similar to that observed in Ago 2 knockdowns (Chotkowski *et al.*, 2008), which would also support an antiviral activity of the piRNA-related pathway in drosophila. More research is needed to determine the possible antiviral activity of piRNAs in drosophila and whether this is restricted to ovary cells or is found in all somatic tissue, and to determine the precise differences between these pathways in aedine mosquitoes and drosophila.

We do not know how the antiviral piRNA pathway is induced in aedine mosquitoes, although previous observations have suggested a dsRNA molecule as the inducer (Morazzani *et al.*, 2012). This would suggest crosstalk between the siRNA and piRNA pathways. A similar result has been reported for *A. gambiae*-derived cells, which show a decrease in dsRNA-induced reporter gene expression following Ago 3 knockdown (Hoa *et al.*, 2003), indicating such crosstalk even in non-aedine mosquitoes. However, the lack of piRNA-like molecules produced in the case of dsRNA transfection alone (Fig. 5) suggests the need for ssRNA (active viral replication) to induce piRNA production. The inhibitory effect observed by the expression of the dsRNA-binding RNAi suppressor B2 by CHIKV on the production of viral-specific piRNAs suggests that this is a secondary effect as dsRNAs are replication intermediates required for ssRNA production (Morazzani *et al.*, 2012). The observation that most piRNAs map to the coding strand region of the 5′ end of the SFV subgenomic mRNA, SINV or CHIKV (Morazzani *et al.*, 2012; Vodovar *et al.*, 2012) suggests that perhaps particular transcripts or genome regions are preferentially targeted. In this case, viral-specific dsRNA, either due to the sequence or structural features such as dsRNA, could be the inducer of the piRNA pathway. Characterization of the viral-specific piRNA-like molecules suggests a ping-pong production mechanism; however, knockdown of Ago 3, which is known to be important for the ping-pong mechanism in drosophila, did not result in an increase of SFV replication. It could be possible that the observed viral-specific piRNA-like molecules are produced in an Ago 3 independent manner in *Ae. aegypti* in contrast to drosophila, or that the obtained knockdown of Ago 3 was not sufficient.

To date, it is not definitively known if the viral-specific piRNA-like molecules in mosquitoes and derived cell lines are really produced through the piRNA pathway using PIWI and Ago 3 proteins, although their ping-pong signature highly suggests this production pathway. In addition, piRNA production models were shown in drosophila using at least two PIWI family proteins for the production of primary and secondary piRNA molecules, but knockdown experiments only showed a strong effect on SFV production for Piwi 4. The low frequency of SFV-specific piRNA-like molecules found in cells with knockdown of all Piwi and Ago 3 proteins strongly supports their involvement in the production of these molecules. However, the lack of decrease in SFV-specific piRNA-like molecules in Piwi 4 knockdowns and the increase in SFV replication and production suggest an effector role of this PIWI-clade Ago protein by using the SFV-specific piRNA-like molecules to target the virus. The observed increase in SFV replication in both Piwi 4 and all Piwi/Ago3 knockdown cells compared to control dsRNA could also explain the increase of 21 nt viRNAs in these cells. In addition, the increase in SFV-specific piRNA-like molecules in combination with a higher SFV replication again suggests that Piwi 4 is not needed for SFV-specific piRNA-like production, but rather it is used to target and thereby silence the virus.

Together, these results show that arbovirus replication is able to trigger the piRNA pathway and that silencing of piRNA-related proteins reduces viral-specific piRNA-like molecules and enhances viral replication and production, suggesting an antiviral response by the piRNA pathway. Both the piRNA and exogenous siRNA pathways may act in combination to control viral infections in mosquito cells. Future research is needed to determine the viral inducer molecule of the piRNA pathway and map the involvement of each Piwi/Ago 3 protein in detail. We cannot exclude that some Piwi-clade proteins that are important in viral piRNA-like production have been missed due to either inefficient knockdown or the need of combinational knockdowns, but our results already suggest Piwi 4 as an effector protein. Besides, the proposed linkage between the siRNA and piRNA pathways has yet to be investigated, and it is not yet known if the piRNA and siRNA pathways are restricting different parts of the viral infection (acute versus persistent infection) in mosquitoes. Experiments in the exogenous RNAi pathway knockout cell lines, such as C6/36 (Brackney *et al.*, 2010; Scott *et al.*, 2010), suggest that the piRNA pathway may still be able to control viral infection to some extent on its own; however, further studies are required to fully assess interaction between the pathways.

## Methods

### 

#### Cells, plasmids and virus.

*Ae. albopictus*-derived U4.4 and *Ae. aegypti*-derived Aag2 cells were maintained in L-15 medium supplemented with 10 % FCS and 10 % tryptose phosphate broth at 28 °C. Amplification and titration of SFV (strain SFV4) and the SFV4(3H)-*RLuc* reporter virus and infection of U4.4 and Aag2 cells were performed in a similar way as previously described; infections were performed at growth temperature (28 °C) (Siu *et al.*, 2011). Briefly, viruses were grown in BHK-21 cells in Glasgow minimum essential medium (GMEM) with 5 % FCS and 10 % tryptose phosphate broth at 37 °C with 5 % CO_2_. Virus purified from the supernatant or virus present in supernatant was titrated by plaque assay on BHK-21 cells using an Avicell (0.6 %)/MEM overlay with 2 % FCS. Infection of mosquito cells was performed in L-15 medium with 10 % FCS and 10 % tryptose phosphate broth for 1 h at 28 °C, followed by a washing step with PBS and overlay with media.

#### Reverse transcription and PCR.

RT-PCR was performed with total RNA (500 ng) isolated using TRIzol (Invitrogen), Superscript III and oligo-dT primer, according to the manufacturer’s protocol. Piwi/Ago 3 transcripts were detected and amplified by PCR (2 µl of the cDNA reaction) using primers containing T7 RNA polymerase promoter sequences (Table S1). For the detection of the transcripts, 40 rounds of PCR using KOD polymerase were performed in contrast to 35 rounds for semi-quantitative PCR using GoTaq polymerase. The eGFP-derived PCR product was produced by using eGFP-C1 (Clontech) as a template. PCR products were gel-purified and used for dsRNA production or first cloned into the pJet blunt 1.2 vector (Fermentas) and sequenced.

#### *In vitro* dsRNA transcription.

dsRNA molecules for Piwi/Ago 3 and eGFP were produced with a T7 RNA polymerase *in vitro* transcription kit (Megascript RNAi kit, Ambion) using a PCR product as a template, followed by column purification. Internally fluorescently labelled eGFP-specific dsRNA was produced in the same way but using fluorescein-labelled rNTP mix (Roche) following the manufacturer’s protocol, and purified by ethanol precipitation. Primer sequences are indicated in Table S1.

#### Cell viability assay.

Viability of cells transfected with dsRNA molecules was determined using a CellTiter-Glo luminescent cell viability assay (Promega) following the manufacturer’s recommendations.

#### Luciferase assay.

Luciferase activities were determined using a Dual Luciferase assay kit (Promega) on a GloMax luminometer following cell lysis in Passive Lysis Buffer.

#### Transfection.

Aag2 cells (1.7×10^5^ per well) were seeded in 24-well plates, 24 h before transfection. Piwi/Ago 3 transcripts were silenced by the transfection of 100 ng dsRNA per well (Piwi specific or 400 nt eGFP) at 24 h post-seeding with Lipofectamine 2000 (Invitrogen), following the manufacturer’s protocol. At 24 h p.t., cells were either harvested to isolate RNA for RT-PCR or infected with SFV4(3H)-*RLuc* at the indicated m.o.i. Supernatant from infected cells (m.o.i. of 0.1) was used to determine virus titre by plaque assays on BHK-21 cells. In addition, luciferase expression was measured 48 h p.i. as described above.

#### Small RNA isolation and sequencing.

Small RNA sequencing was carried out by ARK-Genomics (The Roslin Institute, University of Edinburgh) and The GenePool (University of Edinburgh) using the Illumina Solexa platform. Approximately 5×10^5^ U4.4 cells and 6×10^5^ Aag2 cells per well were transfected in a 6-well plate with 1 µg eGFP-derived dsRNA (720 nt) or left untreated.

For the infection experiments, Aag2 cells were transfected with 1 µg Piwi 4 or eGFP dsRNA or 200 ng each Piwi1-3, 4, 5, 6, 7 and Ago 3 dsRNA using Lipofectamine 2000. At 24 h p.t., cells were infected with SFV4 at an m.o.i. of 10. At 24 h p.t. or p.i., RNA was isolated using 1 ml TRIzol (Invitrogen) per well, followed by purification, sequencing and analysis as previously described (Schnettler *et al.*, 2013).

#### Immunostaining.

Aag2 cells were fixed in formaldehyde and permeabilized by 0.3 % Triton/PBS for 30 min, followed by a wash with PBS. Cells were pre-incubated with CAS-Block for 1 h at room temperature, followed by incubation with CAS-Block diluted SFV nsP3-specific antibody (1 : 500) (Siu *et al.*, 2011) for 90 min at room temperature. After three washing steps with PBS, an anti-rabbit antibody conjugated with Alexa Fluor 543 diluted in CAS-Block (1 : 1000) was incubated for 60 min at room temperature. Following further washing steps with PBS, cells were dried and mounted with DAPI-containing hard set Vectashield mounting medium (Vector Laboratories), and fluorescence was detected on a Zeiss LSM Meta microscope.
